# SPECTRE—A novel dMRI visualization technique for the display of cerebral connectivity

**DOI:** 10.1002/hbm.25385

**Published:** 2021-02-27

**Authors:** Marco Reisert, Christoph P. Kaller, Marvin Reuter, Horst Urbach, Bastian E. Sajonz, Peter C. Reinacher, Volker A. Coenen

**Affiliations:** ^1^ Department of Stereotactic and Functional Neurosurgery Freiburg University Medical Center Freiburg Germany; ^2^ Medical Faculty of Freiburg University Freiburg Germany; ^3^ Department of Radiology Medical Physics, Freiburg University Medical Center Freiburg Germany; ^4^ Department of Neuroradiology Freiburg University Medical Center Freiburg Germany; ^5^ Center for Deep Brain Stimulation Freiburg University Freiburg Germany

**Keywords:** brain, deep brain stimulation, diffusion MRI, HARDI, midbrain, neurosurgery, stereotactic navigation, subthalamic nucleus, tractography, visualization

## Abstract

The visualization of diffusion MRI related properties in a comprehensive way is still a challenging problem. We propose a simple visualization technique to give neuroradiologists and neurosurgeons a more direct and personalized view of relevant connectivity patterns estimated from clinically feasible diffusion MRI. The approach, named SPECTRE (Subject sPEcific brain Connectivity display in the Target REgion), is based on tract‐weighted imaging, where diffusion MRI streamlines are used to aggregate information from a different MRI contrast. Instead of using native MRI contrasts, we propose to use continuous template information as the underlying contrast for aggregation. In this respect, the SPECTRE approach is complementary to normative approaches where connectivity information is warped from the group level to subject space by anatomical registration. For the purpose of demonstration, we focus the presentation of the SPECTRE approach on the visualization of connectivity patterns in the midbrain regions at the level of subthalamic nucleus due to its importance for deep brain stimulation. The proposed SPECTRE maps are investigated with respect to plausibility, robustness, and test–retest reproducibility. Clear dependencies of reliability measures with respect to the underlying tracking algorithms are observed.

## Graphical abstract

SPECTRE (Subject sPEcific brain Connectivity display in the Target REgion) is based on tract‐weighted imaging, where diffusion MRI streamlines are used to aggregate information from a different MRI contrast. Instead of using native MRI contrasts, we propose to use continuous template information as the underlying contrast for aggregation. The connectivity patterns in the midbrain as one of most important brain regions for stereotactic neurosurgery serve as an application example of the proposed technique.

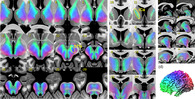

**Abbreviations**
DBS: deep brain stimulation
DLPFC: dorsolateral prefrontal cortex
DMPFC: dorsomedial prefrontal cortex
dMRI: diffusion magnetic resonance imaging
FACT: fiber assignment by continuous tracking
fMRI: functional magnetic resonance imaging
ICa: Internal capsule, anterior limb
MRI: magnetic resonance imaging
MSR: medial STN region
OCD: obsessive compulsive disorder
OFC: orbitofrontal cortex
SPECTRE: Subject sPEcific brain Connectivity display in the Target REgion
STN: subthalamic nucleus
T2W: T2 weighted
UPDRS: Unified Parkinson's Disease Rating Scale
VAT: volume of activated tissue
VTA: ventral tegmental area
VMPFC: ventromedial prefrontal cortex

## 1 INTRODUCTION

The analysis and visualization of the brain's white matter structures by diffusion magnetic resonance imaging (dMRI) is becoming an important prerequisite for planning neurosurgical interventions (Essayed et al., 2017). Tractography allows a preoperative, noninvasive localization of functionally important and interventionally relevant fiber trajectories. However, several issues are still hampering tractography to be widely implemented. On the one hand, reliability, validity, and the dependency on the actual tractography method are limiting factors. On the other hand, the actual selection protocols for the segmentation of bundle structures relevant in the context of a given intervention are cumbersome to specify and depend on the actual planning systems used (Rheault et al., 2019). Particularly in the midbrain region, on which we will concentrate in the following, there is not even an agreed on and established taxonomy of bundles due to the complex fiber anatomy. As a direct consequence, the explicit white matter bundle segmentation is per se not well defined in the midbrain region. Nevertheless, especially with respect to planning deep brain stimulation (DBS) surgery in this region, it is of interest to characterize the projective fields of the stimulated structures on the individual level.

To address at least parts of these problems here we propose a rather simple, but generalized approach to visualize structural connectivity information in certain target regions, which can guide neurosurgeons during their planning process. In the following, we will refer to this novel approach as SPECTRE—Subject sPEcific brain Connectivity display in Target REgion.

Practically, SPECTRE may be seen as an instance of track‐weighted imaging (Calamante, 2017; Calamante et al., 2012) (TWI). In TWI streamlines are used to average nonlocal information stemming from a different, not necessarily dMRI‐related contrast (like T2W MRI or fMRI). The value of a certain voxel is determined by (a) seeding streamlines in that voxel, and by (b) using these streamlines to aggregate the foreign contrast along the streamlines and to use the aggregated quantity as the image value at the seeding location. The simplest way to aggregate the information along the streamline is averaging/summation. However, other operations such as max pooling are possible. Instead of native anatomical contrasts, we propose to use normative geometric information as the underlying contrast for aggregation. That is, the information is stemming from a certain template space (such as the MNI standard space), which was previously registered to the native patient space. For example, if we want to know whether a voxel in the brain is connected to the anterior rather than the posterior brain, we just compute the mean of the MNI *y*‐coordinate along each streamline connected to this voxel. Repeating this for every voxel in a target region of interest can thus give an intuitive understanding of the local connectivity patterns.

Previous work is partly related to the proposed methodology. SPECTRE is a special type of TWI (Calamante, 2017; Calamante et al., 2012) with a particular choice of contrast. Similar ideas have been used for tract driven segmentation (Behrens et al., 2003; Behrens et al., 2007; Ewert et al., 2018; Safadi et al., 2018), where certain regions of interest stemming from atlas spaces are used as “contrast” for aggregation. In fact, those approaches may also be regarded as a special type of TWI, where the aggregation operation is related to a maximum operation. However, SPECTRE does not provide binary segmentation decisions, but direct, continuous contrasts helping surgeons to navigate and validate their decisions. Other work close to our approach has been reported by (Barrick et al., 2009) who used coordinate information from template space to produce certain colorings. In addition, they introduced a special eigenvalue decomposition of the coordinates of the terminal positions of the streamlines to join information from both ends from the streamline.

In the following, we will introduce SPECTRE by concentrating on the connectivity patterns in the midbrain as one of most important brain regions for stereotactic neurosurgery in the context of deep brain stimulation, typically performed in the subthalamic nucleus (STN) for Parkinson's disease. As an additional example for demonstration, we will consider the internal limb of the internal capsule (ICa) which has become an investigational target for psychiatric disorders such as obsessive compulsive disorder (OCD) and major depression (MD).

## 2 MATERIALS AND METHODS

### 2.1 Imaging

We demonstrate SPECTRE on three different types of dMRI datasets: First, we consider high quality dMRI data based from the Human Connectome Project (HCP). In addition, we apply SPECTRE to test–retest data from normal controls measured with a clinically feasible dMRI sequence on a Siemens TIM Trio to analyze its reliability. Finally, we will show the clinical utility of SPECTRE maps based on a single dataset from an example patient case, which was measured on a Siemens TIM Prisma.

#### 2.1.1 HCP

Data from the Human Connectome Project database (https://ida.loni.usc.edu/login.jsp), Q1: S3, S4, were used. Overall, 200 normal subjects (78 male, mean age ± SD, 29 ± 3.5 years) were analyzed (resolution 1.25 mm isotropic, three *b*‐shells with 1,000, 2000, 3,000, for more details on the protocol and preprocessing see (Glasser et al., 2013).

#### 2.1.2 TRIO

Twenty‐six normal subjects were scanned on a Siemens TIM TRIO using a 1‐shell protocol with *b*‐value 1,000 and 60 directions per shell, at an isotropic resolution of 2 mm, 6/8 partial Fourier, TR = 10,900 ms, TE = 107 ms. Additionally, a T1‐weighted structural dataset was acquired, resolution 1 mm isotropic. For distortion correction, the PSF‐mapping technique was used (Zaitsev et al., 2004). Each subject was scanned twice (in two different sessions) to investigate the robustness and reliability of dMRI measures.

#### 2.1.3 PRISMA

A patient was scanned on a Siemens 3T TIM PRISMA using an SE EPI sequence with a TE = 88 ms and TR = 2008 ms, bandwidth 1780 Hz, flip‐angle 90, GRAPPA factor 2, SMS factor 3 with 17 nondiffusion weighted images, 2*58 images with *b*‐factor *b* = 1,000 and 2,000 s/mm^2^; with an in‐plane voxel size of 1.5 mm 1.5 mm and a slice thickness of 3 mm. One phase‐encoding flipped *b* = 0 image was acquired, which is used for distortion correction (FSL's topup, Smith et al., 2004). The diffusion weighted images were first denoised by a postprocessing technique which uses random matrix theory (Veraart et al., 2016). This was followed by Gibbs artifact removal based on local sub‐voxel shift (Kellner et al., 2016). Afterwards, images were corrected for EPI distortions by FSL's topup and finally up‐sampled to isotropic resolution by an edge‐preserving interpolation approach (Reisert & Kellner, 2017).

### 2.2 The SPECTRE method

**FIGURE 1:**
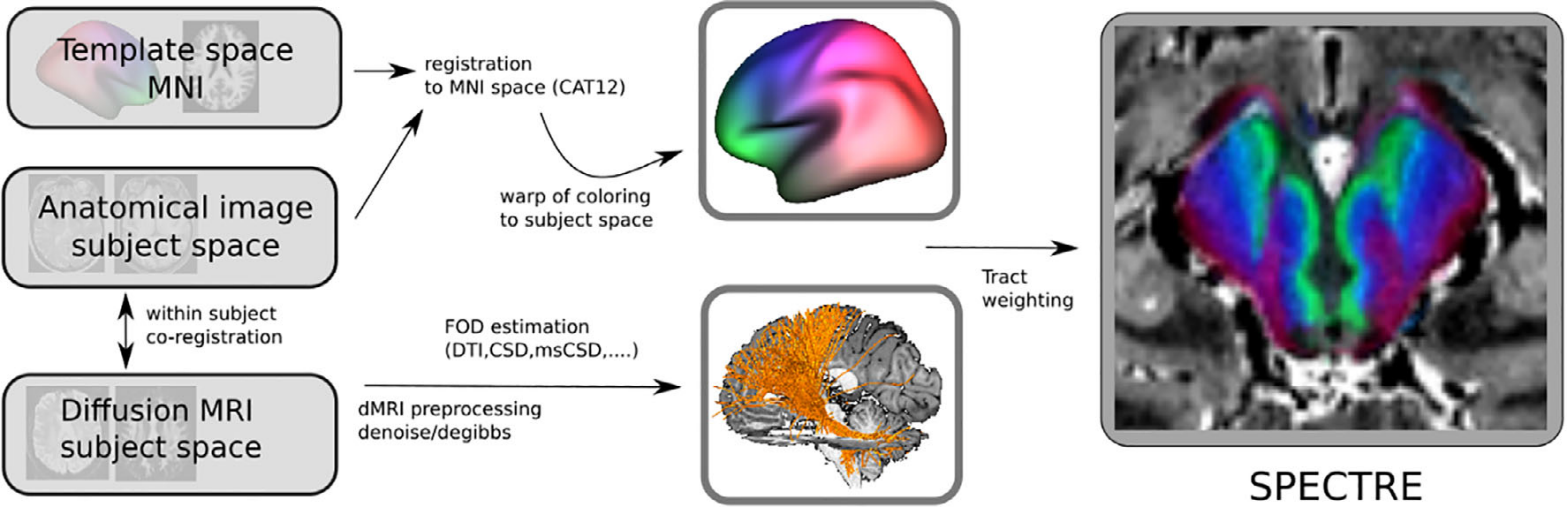
Processing flow of the approach: A color‐coding scheme defined in MNI space is warped to subject space and used for generating SPECTRE maps by a tract weighting approach

The general idea is visualized as a flowchart in Figure 1: In a first step, we coregister the individual anatomical MRI (like T1 and T2 weighted) and the dMRI (here by using the coregistration implemented in SPM12; https://www.fil.ion.ucl.ac.uk/spm/software/spm12/). Then, a diffeomorphic mapping between subject space and template space is established (here using CAT12, http://dbm.neuro.uni-jena.de/cat12/CAT12-Manual.pdf). Based on this mapping a task specific color‐coding (how to choose this coding is detailed below) is warped from template to subject space, which is then used as a basis for the generation of tract‐weighted images (TWI) finally resulting in the SPECTRE maps. There actually exist a multitude of different implementations of the TWI procedure itself. For the present purpose of a probabilistic streamline integration/propagation we considered two options: On the one hand, we considered an approach which is close to the tracking algorithm implemented in FSL (Behrens et al., 2007), which may again be based on various options for the estimation of the fiber orientation distribution (see Appendix S1 for details). On the other hand, we considered a more simple approach, namely tensor deflection (Lazar et al., 2003) (TEND), which is solely based on the diffusion tensor and hence being applicable also on (clinical) data of rather poor quality. For the FSL‐based tractography, we considered five options to compute the fiber orientation distribution (FOD) and tracking directions: Principal Tensor Direction (DTI), Constrained Spherical Deconvolution (CSD) according to (Tournier et al. 2007; Tournier et al. 2013), multishell CSD (msCSD) according to (Jeurissen et al, 2014; Dhollander et al., 2018), Fiber Continuity Regularized CSD (fcCSD) according to (Reisert et al, 2011, Reisert & Skibbe, 2013). In addition, we computed tract orientation distributions (TODs) based on Global Tractography (Reisert et al., 2011) (GT), for details see Appendix S1.

To generate the final SPECTRE contrast an integration of the color‐coding scheme along the streamline is applied, similar to the TWI procedure as described in Calamante et al. (2010). To be more precise: let *c*_*k*_ be the kth channel of the color scheme under consideration (already transformed to subject space), and suppose we have emitted *N* streamlines at position (seed) *r* within the volume of interest according to some tracking scheme as described above. Each of these streamlines is represented by a series of coordinates $\left(x_{1}^{r,f},x_{2}^{r,f},x_{3}^{r,f},x_{4}^{r,f},\ldots \right)$, where the two upper indices refer to seeding position and the streamline number, the lower index to the sampling point of the streamline. The SPECTRE map is then formed by

$$C_{k}\left(r\right)=\sum_{f=0}^{N-1}\sum_{n=1}^{L_{f}}c_{k}\left(x_{n}^{r,f}\right)$$

where the outer sum runs over all streamlines seeded at the position *r* and the inner sum runs over the parametrization of the streamline. The contrasts are evaluated at fractional positions *x*_*n*_ by simple trilinear interpolation. Note that the sum is not normalized by the length of the streamline, that is, longer streamlines contribute more than short ones. However, for visualization we have to apply a normalization scheme, as color channels have to fall within a fixed [0,1] range. There is no straightforward way to normalize SPECTRE maps so that brightness can be visualized without additional windowing. To automatically choose a visually appealing brightness and contrast we took the following approach: we computed the brightness (sum of individual color channels) and divided the SPECTRE maps by the 80% percentile of all brightness values within the considered target region. As there can be a few “overexposed” values (a single channel has values above 1), we simply clipped these values to the valid range. Note that this normalization was only applied for visualization purposes. For quantitative comparisons, such as the reproducibility analysis or any other subsequent analysis, no prior normalization was applied.

### 2.3 Generation of the fronto‐occipital color gradients

**FIGURE 2:**
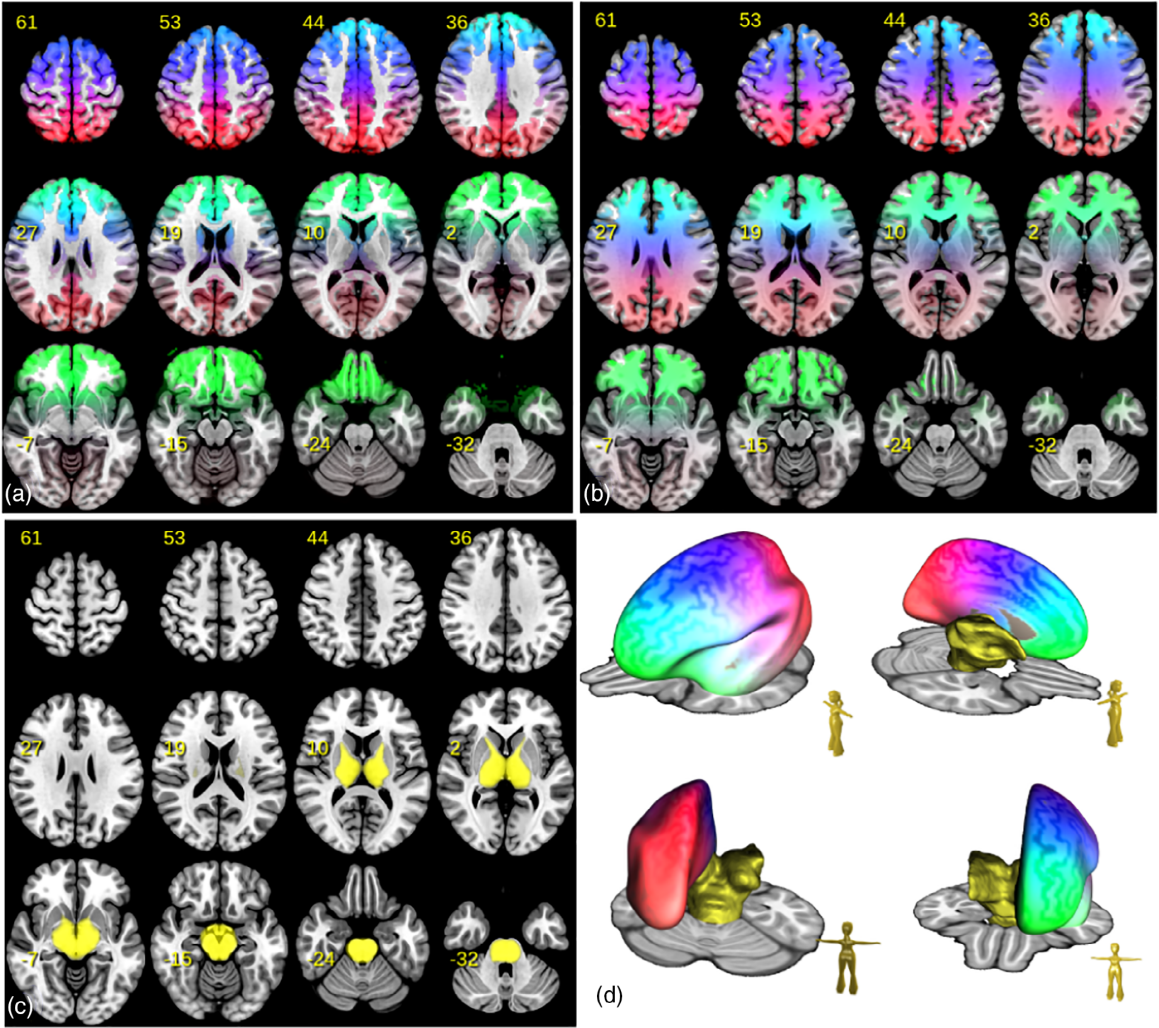
Fronto‐occipital color gradients: Images (a) and (b) show the color gradient in an axial mosaic overlayed on a T1 image in MNI template space. Additionally, in (d) the coloring is shown in 3D projected onto an inflated MNI brain surface together with the midbrain region considered for evaluation, which is also shown in (c) as mosaic

To warp information from normative space (in our case MNI space) we relied on CAT12 (http://dbm.neuro.uni-jena.de/cat12/CAT12-Manual.pdf) using the Statistical Parametric Mapping software (SPM12, http://www.fil.ion.ucl.ac.uk/spm/software/spm12). We used a simple continuous coloring scheme of the MNI brain ranging from green for prefrontal regions to blue containing sensory/motor regions, and ending up in red for the occipital lobe (see Figure 2). We used the following formula

$$c\left(r\right)=\left[c_{1}\left(r\right),c_{2}\left(r\right),c_{3}\left(r\right)\right]=\sum_{i=1}^{3}a_{i}\exp\left(-,{\left|r_{i}-r\right|}^{2},/,\left({2\sigma }^{2}\right)\right)$$

to generate the colored volume. The components *C*_*k*_ refer to colors, namely red—*c*_1_, green—*c*_2_ and blue—*c*_3_. The position *r* refers to the MNI coordinate in template space.The specific parameters were chosen to be *a*_1_ = [0.5 0 0], *r*_1_ = [0 − 60 70], *a*_2_ = [0 1 0], *r*_2_ = [0 70 00], *a*_3_ = [0 0 1], *r*_3_ = [0 20 70] and *σ* = 50. The rationale of this coloring scheme was developed in appreciation of deep brain stimulation (DBS) planning especially for the subthalamic nucleus (STN). STN anatomy typically is interpreted as follows: Medial and anterior parts of the nucleus are regarded as “limbic” and connect to prefrontal and frontopolar parts of the cortex. Adjacent and more posterior regions are the prefrontal association regions followed on the posterior and lateral aspect by motor parts (Haynes & Haber, 2013). We therefore decided to color code frontopolar, orbitofrontal regions and then prefrontal and associative regions followed by motor and then sensory regions resulting in the fronto‐occipital color gradient applied to the cortex. With this strategy, the STN was color‐coded in a “fronto‐polar to motor” gradient (green to blue) by its mere connectivity pattern.

Another question is where to accumulate the coloring: One could accumulate just at the cortical destination, or one could already start accumulation in the white matter. In Figure 2a,b, we show the proposed coloring restricted on either gray matter or white matter, which are the two schemes we used here.

To demonstrate the effectiveness of the proposed visualization technique we concentrated on the midbrain, basal ganglia, and parts of the anterior limb of the internal capsule. Thus, the region where Equation (5) is computed is the region depicted in Figure 2c in yellow.

### 2.4 Parameter determination

To understand the influence of the different involved parameters, different settings were evaluated. One parameter is the number of streamlines seeded per voxel. We selected this parameter mainly by qualitative visual inspection and found that the stability of the estimation is mainly influenced by the underlying orientation distribution used for propagation. For example, for a rather tight distribution of streamlines, which typically appear for DTI based orientations, fewer seeds are necessary to obtain “clean” maps. Nevertheless we decided to use a fixed number for all methods for which 500 seeds per voxel seemed to be a reasonable trade‐off between quality and running time. One further parameter is the voxel size of the final SPECTRE maps. Depending on the quality of the raw data, we decided for 0.5 mm isotropic resolution for the HCP and PRISMA datasets and a coarser resolution of 1 mm isotropic for the TRIO dataset. Note that the resolution is approximately twice the original imaging resolution, which is in concordance with other related techniques (Calamante et al., 2010), where similar or even higher super‐resolution factors are achieved. Indeed, running time cannot be neglected: in the high‐resolution setting (0.5 mm) approximately 100 million streamlines have to be tracked, which takes on a workstation (Intel I7, RAM 32GB), single core, a few hours. By restriction of the target region and sacrificing resolution (e.g., 1 mm isotropic), and parallel computation, the computation time can be turned down to below a minute. There are two other parameters crucial for the appearance of the SPECTRE maps: the step width *α* and the noise factor *s* used to perturb the propagation direction (see Appendix for details). Both parameters are mutually dependent. To limit the space of possibilities we decided to use a fixed step size of *α* = 1 mm, which is appropriate in the view of the dMRI imaging resolutions (1.25 mm for HCP, 1.5 mm for PRISMA and 2 mm for TRIO). The noise factor was varied between *s* = 0.05 − 0.2.

### 2.5 Re‐scan reproducibility

A major prerequisite for any kind of visualization technique for neurosurgical planning is the reproducibility of the results. The TRIO dataset consisted of 26 subjects scanned twice on the same scanner, but in different sessions. This enabled us to compute reproducibility measures for the different SPECTRE approaches. To do the comparison, the SPECTRE maps were normalized to common MNI reference space (at 1 mm resolution) by the diffeomorphic mappings provided by CAT12. Then, they were compared with respect to stability by the intraclass distances relative to interclass distances. A class is here synonymous for a subject. For each subject there are several measurements (here SPECTRE maps), and we quantified the distances between SPECTRE maps from the same subject but different scans relative to the distances between SPECTRE maps from different subjects. More precisely, we computed the following measure

$$\mathrm{icd}=100\%\left(1-N\;\sum_{n=1}^{N}{\left|s_{n}^{1}-s_{n}^{2}\right|}^{2}/\sum_{n=1}^{N}\sum_{j=1}^{N}{\left|s_{j}^{1}-s_{n}^{2}\right|}^{2}\right)$$

where $s_{n}^{k}$ denotes the SPECTRE map of the *n*‐th subject for the *k*‐th scan (*k* = 1,2). The color components of the SPECTRE map were treated equally and the Euclidean distance is calculated over all voxels within the volume of interest. The *icd* index ranges between 0% and 100% giving smaller values for nonreproducible maps and 100% for highly reproducible maps.

### 2.6 Case example

To illustrate the clinical utility of SPECTRE maps we show a single case of STN DBS (Vercise Gevia DBS system with Cartesia directional leads, Boston Scientific, VA, CA) stimulation adjustment in a 59‐year‐old patient suffering from advanced Parkinson's disease. The patient had preoperative dMRI and anatomical T1w and T2w scans. SPECTRE maps were overlaid with anatomical T2w scan to allow registration. Electrode locations were extracted from postoperative CTs. Electrode rotations were determined with Guide XT® (Boston Scientific & BrainLab, Munich, Germany) from postoperative CT and additionally checked with routine rotational fluoroscopy (Reinacher et al., 2017). The stimulated sites were simulated as a volume of activated tissue (VAT), using Guide XT® Elements (BrainLab). The VAT describes the volume, where the probability that axons are activated due to electrical stimulations of the electrodes is significant. For integration with SPECTRE, the maps were overlaid on the preoperative T2 contrast and transferred via DICOM to Guide XT®/BrainLab elements system (a,b).

### 2.7 Looking with SPECTRE at the ICa

The primary focus of the present report was laid on the midbrain, but SPECTRE may also be applied to other brain regions. SPECTRE is particularly suited to visualize connectivity patterns of projection systems where source and target areas can be clearly defined, for example, the thalamic and other deep gray matter nuclei. But also passage‐ or bottleneck‐like regions are possible areas to look at. As a further example, we therefore demonstrate the connectivity patterns of the anterior limb of the internal capsule (ICa) which serves as a pass‐through of fibers projecting from and towards the prefrontal cortex (Coenen et al., 2020; Nanda et al., 2017; Safadi et al., 2018). Figure 9 provides an illustrative example of the ICa as the only target area with the color labeling being restricted to the prefrontal cortex.

## 3 RESULTS

**FIGURE 3:**
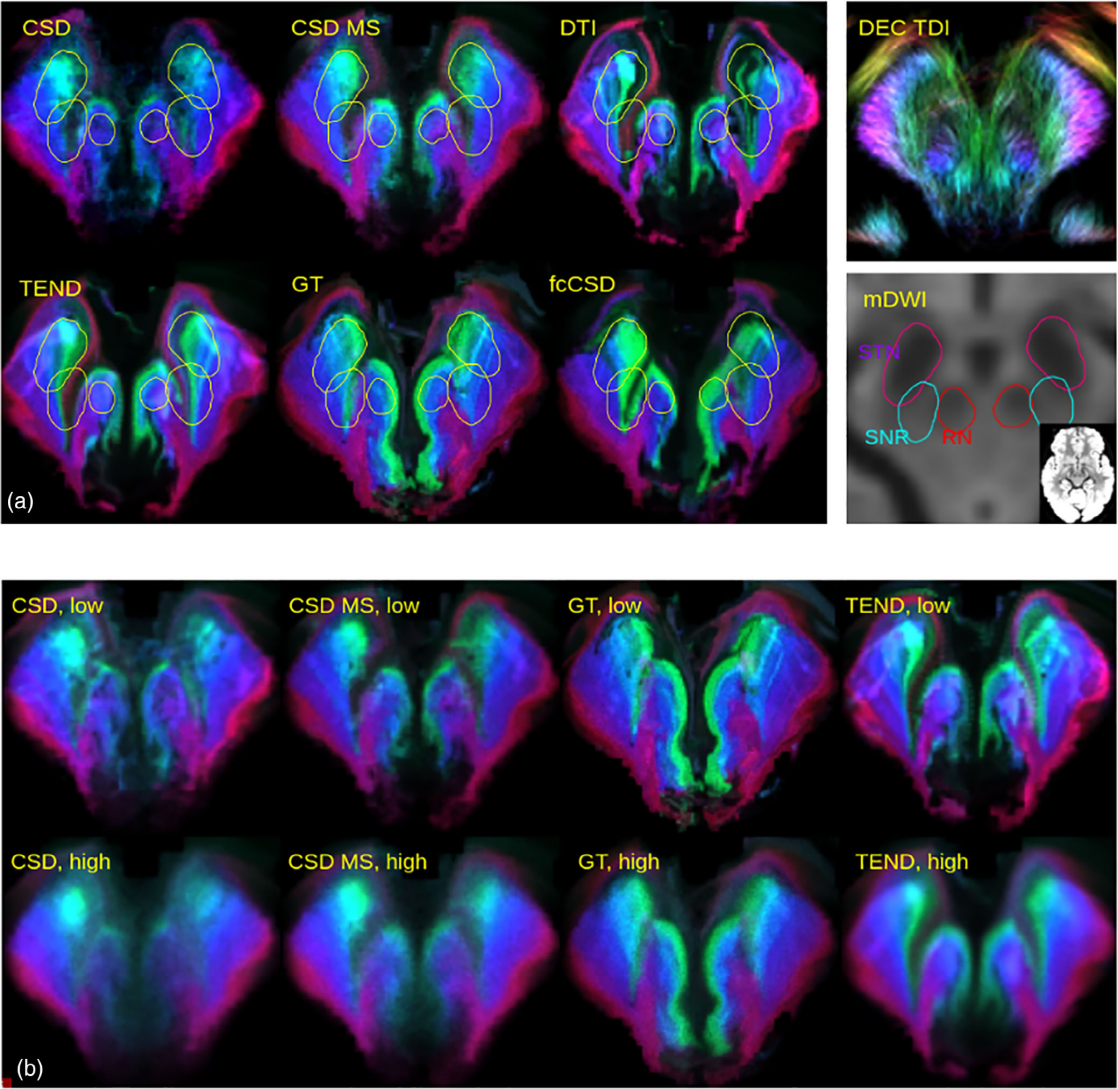
Results for an example dataset from HCP data. In (a) the effects of the different types of direction estimation on the SPECTRE maps are demonstrated. For reference color‐coded tract density images (DEC TDI) are also shown. The maps are shown at an axial slice (at approx. MNI *z* = −8) for an HCP subject (ID = 185,442) zoomed to the midbrain. To understand anatomy also the mean diffusion weighted image is shown (excluding *b* = 0) together with the approximate location of the Substantia Nigra (SNR), Red Nucleus RN) and Subthalamic Nucleus (STN). In (b) the CSD, multi‐shell CSD, GT and TEND are compared for the same slice with low noise (*s* = 0.05)and high noise (*s* = 0.2) levels

We start with a qualitative comparison of the different methods for the determination of the tracking direction (GT, CSD/MS, fcCSD, DTI). The coloring used is the white matter coloring as shown in Figure 2b and the noise factor is *s* = 0.05. In Figure 3a, the SPECTRE maps are shown at the axial level relevant for DBS of the STN. Additionally, outlines of the STN, RN and SNR are shown as obtained from the atlas by Ewert et al. (2018), and color‐coded tract density images (Calamante et al., 2011) (DEC TDI) based on the GT are also given for reference. Unconstrained single‐shell CSD shows rather chaotic behavior, in particular in regions with low anisotropy. Multi‐shell CSD, DTI, fc‐constrained CSD, and GT show a more coherent behavior, but there are qualitative differences. One may use the symmetry of the maps as a proxy for stability. In this respect TEND, GT and CSD MS seem to perform comparably well, while the DTI approach is the most unstable. When comparing SPECTRE with DEC TDI the differences are obvious.

Figure 3b shows a comparison of CSD/MS, GT, and TEND with respect to the noise parameter *s* at 0.05 and 0.2. The edges and contrast of the GT SPECTRE maps are well preserved under different noise levels, while for single‐shell CSD the higher noise levels make the appearance of the SPECTRE maps rather uniform.

**FIGURE 4:**
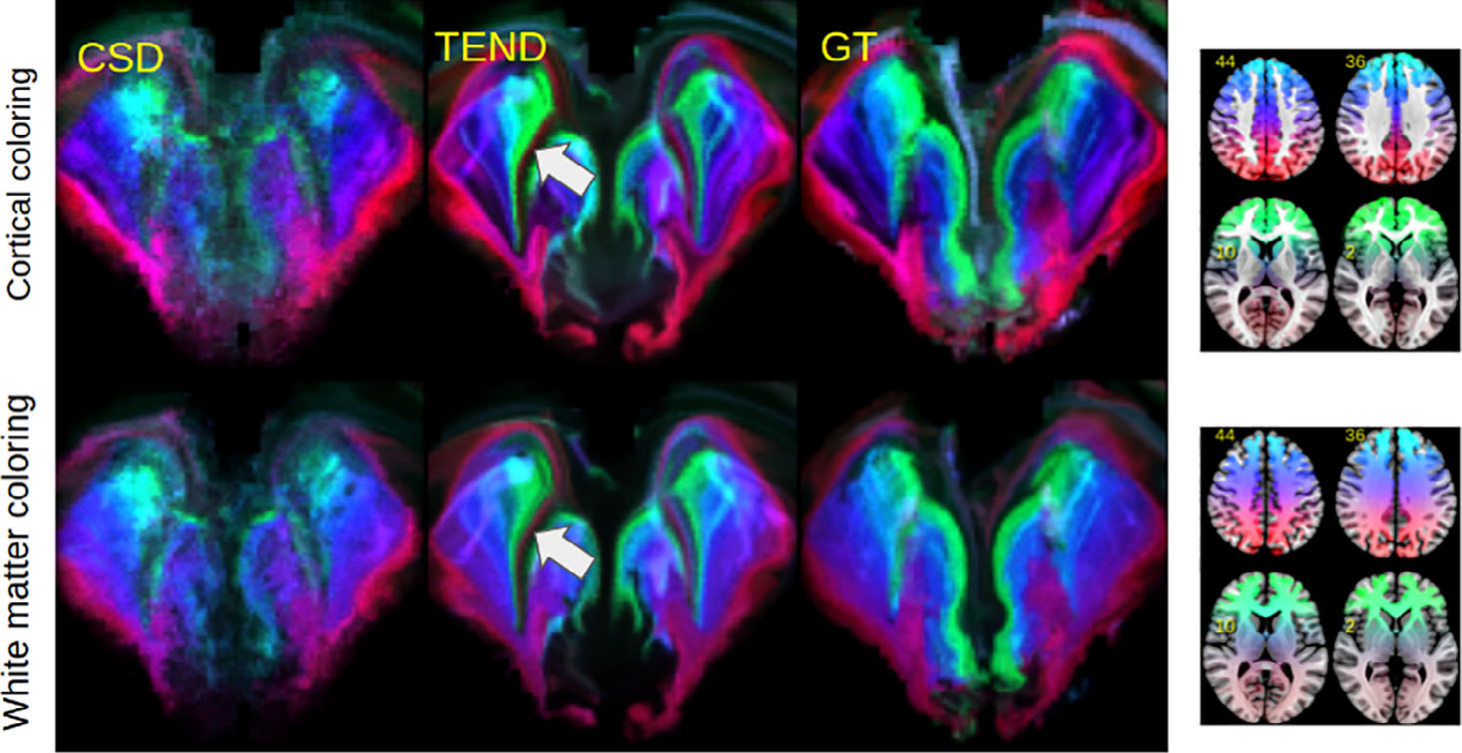
Comparison on the HCP dataset for a cortical coloring scheme (top) versus white matter coloring (bottom) for three different methods (CSD, TEND, GT). The streamlines seeded in dark regions indicated by the arrows are jumping onto the anterior‐commissure and/or terminating in gray matter regions around the Pallidum

Figure 4 shows a comparison of the two different coloring schemes proposed. Obviously, the differences are relatively small compared to differences between the methods for orientation estimation. It is not too surprising that a cortical coloring and a white matter coloring give similar results, because the streamlines emitted in the midbrain are usually rather straight and show a starlike pattern. That is, the path within white matter can predict quite well where the streamline will terminate. One has to note the black stripes marked by the arrows in Figure 4. The effect occurs often for the DTI based approaches, but also sometimes for fcCSD and CSD. We found that most of the streamlines starting in this region are leaping up the anterior‐commissure and/or terminating in gray matter regions around the Pallidum. That is, those streamlines are rather short and due to the implicit tract length weighting, the regions appear as dark voids in the SPECTRE maps. The GT‐based maps show this phenomenon quite occasionally, which suggest that GT copes differently with the region where the anterior commissure is touching/crossing the prefrontal midbrain projection pathways.

**FIGURE 5:**
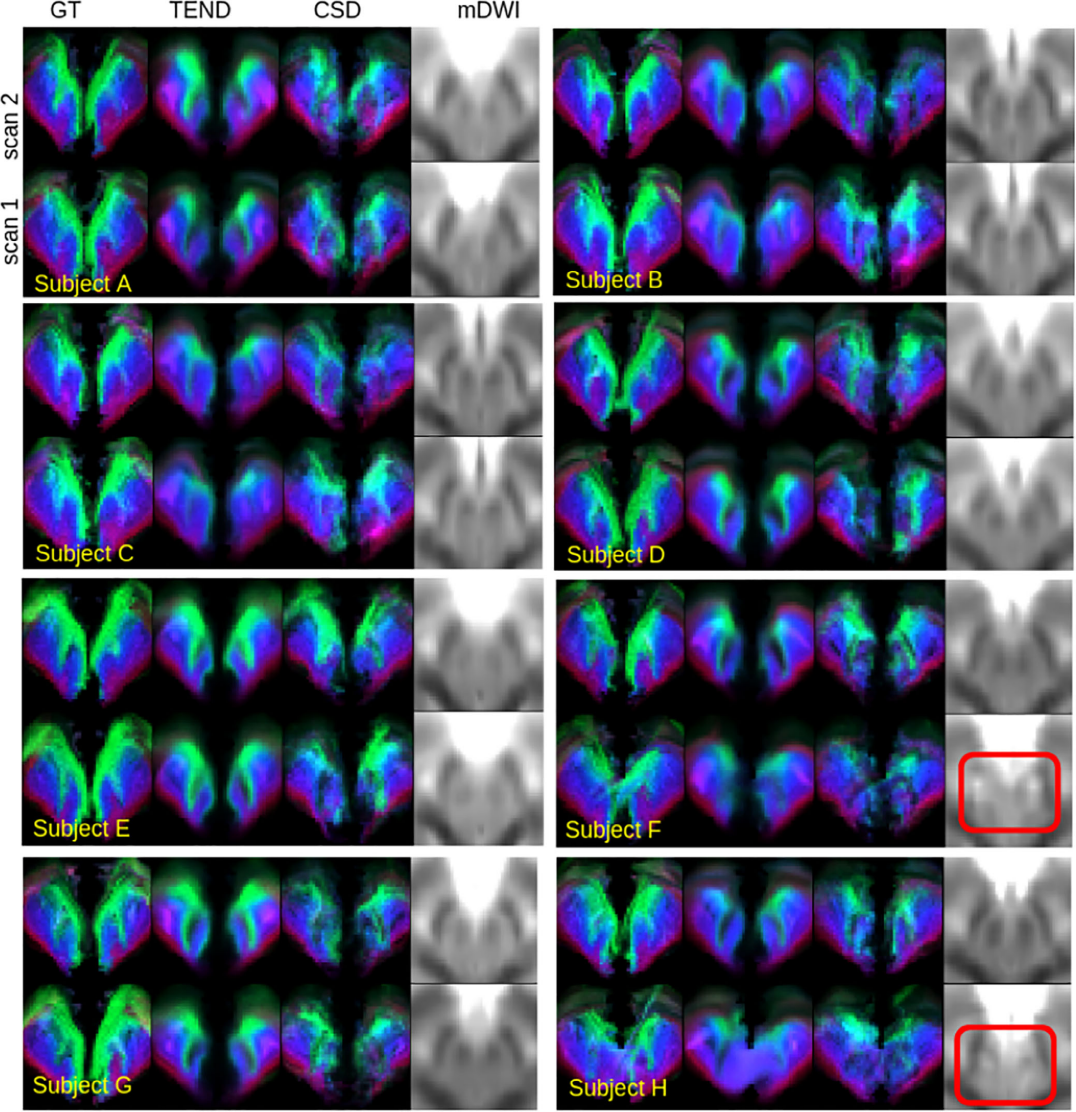
Qualitative reproducibility on the TRIO dataset for eight different subjects at the axial slice *y* = −8 in MNI space. Comparing the methods GT and TEND show acceptable results, while CSD shows rather poor performance. While subjects (a–e) show relatively stable SPECTRE maps across scans, subject (f) and (h) show strong distortion artifacts for its second scans, which are already visible in the mDWI. The red nuclei (the two central black dots) and the STN (black regions lateral to RN) are heavily disrupted, marked by the red rectangles. The distortions have also a large impact on the SPECTRE maps

The between‐session test–retest reproducibility is qualitatively inspected in Figure 5 for CSD, TEND and GT by showing SPECTRE maps at level *y* = −8 in MNI for 8 subjects and both scans of the TRIO dataset. Additionally, we show the mean of all diffusion‐weighted images for reference (mDWI), which shows the susceptibility imprint of the nuclei and may serve as a marker for the EPI‐related image distortions. Subjects A‐E do not show heavy image distortions, and hence, the appearance of the GT and TEND SPECTRE maps is quite consistent. However, CSD shows strong problems to deliver stable results. On the other hand, subjects F and H have suffered from rather strong susceptibility induced distortions which are also reflected by all SPECTRE maps. To quantify the reproducibility, we computed the intraclass distance metric (icd), which tells how well one can distinguish different subjects based on their SPECTRE maps. As already Figure 5 is suggesting, TEND is the most robust approach with the largest icd of 59%, followed by GT (42%), fcCSD (41%) and a rather poor performance for CSD (29%).

**FIGURE 6:**
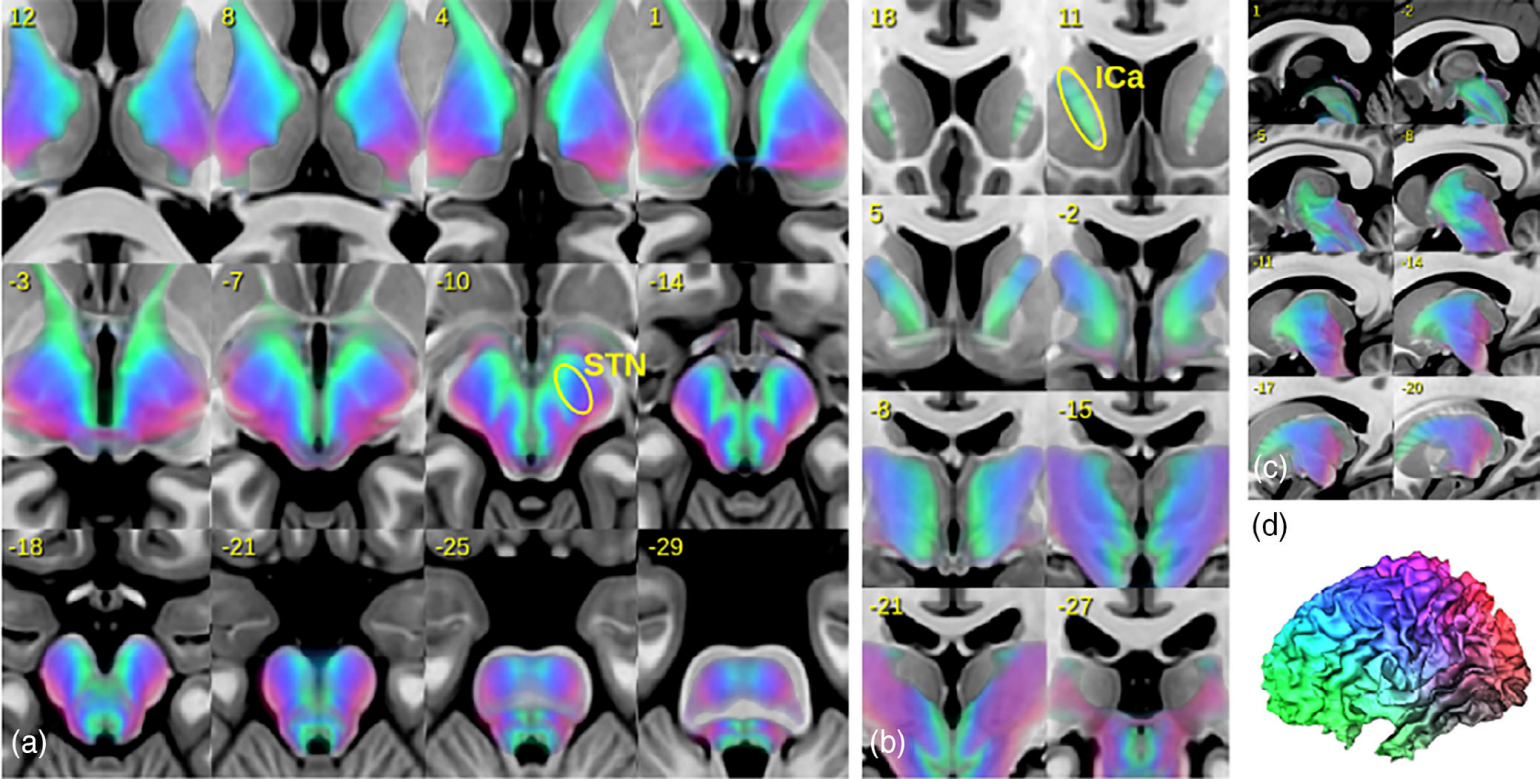
Illustration of group‐averaged SPECTRE maps in MNI space. In (a–c) axial, coronal and sagittal slices are shown. In (d) the associated cortical coding is shown. Legend: ICa, anterior limb of internal capsule; STN, subthalamic nucleus

**FIGURE 7:**
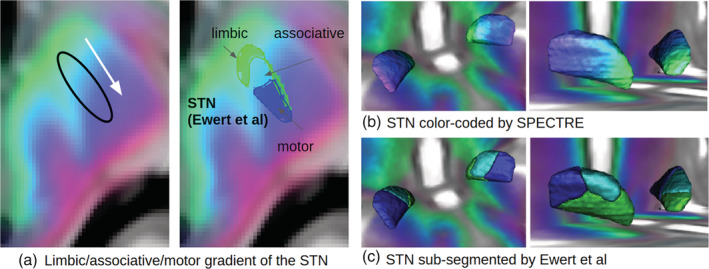
SPECTRE maps in MNI space compared to sub‐segmentations (limbic, associative, and motor) of the STN as proposed in Ewert et al. (2018). A close‐up of a single axial slice is shown in (a). The 3D surface plots show the STN either color‐coded by SPECTRE (b) and the corresponding atlas segmentation (c)

Finally, we compared the histological and tractographically derived STN sub segmentation as proposed by Ewert et al. (2018) with SPECTRE. Therefore, we transformed GT‐based SPECTRE maps produced on the HCP dataset to MNI space and produced a group average. Figure 6 shows the SPECTRE average for different slicings, while Figure 7 shows a close‐up comparison of the limbic/associative/motor classification as proposed in Ewert et al. (2018) with the proposed colorings. The agreement is obvious: the frontopolar, orbitofrontal (green) regions can be regarded as limbic; next to the prefrontal and associative regions (cyan, as a mixture of green and blue) and finished by motor and sensory regions (blue). Slight differences are observable for the frontolateral and inferior margin of the STN, where Ewert's segmentation shows more limbic compounds, while SPECTRE has more motor connotations.

**FIGURE 8:**
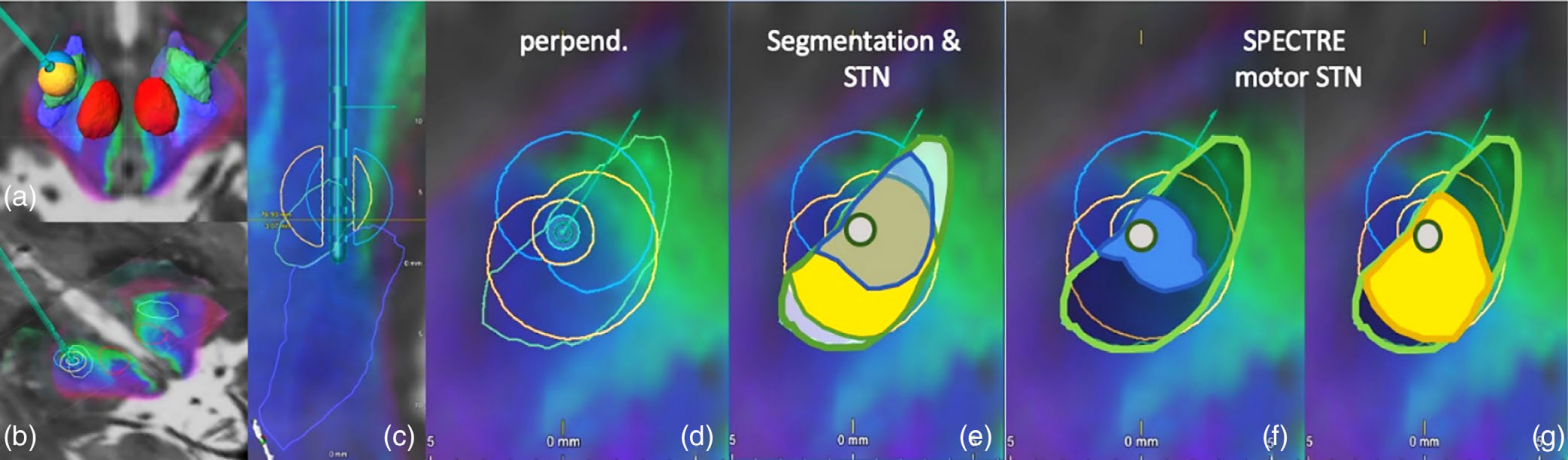
Illustration of SPECTRE application in a single case of subthalamic nucleus (STN) deep brain stimulation (DBS). Only the left side is shown as an example: (a–c) principal electrode position. (c) Sagittal, segmented electrode lead in place. STN green outline. (d,e) Reconstruction of “volume of activated tissue” perpendicular to DBS electrode, blue outline initial electric field, yellow final and rotated electric field. (e) Rotated yellow covers more of general STN than blue stimulation field. (f–g) SPECTRE identifies “motor” STN (blue background), the yellow field covers significantly more “motor” STN corresponding to short term improved outcome

*Results from our example case* are shown in Figure 8. The VAT was rotated based on imaging and simulations in order to cover more sensory‐motor STN (SPECTRE “blue”). As a result, improvements were seen in motor performance in the short term as noted on part III of the Unified Parkinson's Disease Rating Scale (UPDRS). Before adjustment: UPDRS III in DBS OFF‐condition 20 to ON‐condition of 14, still showing motor fluctuations. After Adjustment: UPDRS III OFF‐condition 21, ON‐condition 10, no fluctuations. Despite co‐modulation of limbic (“green”) STN, no immediate psychotropic effects were observed.

## 4 DISCUSSION

In this work, we proposed a novel visualization technique to give clinical neuroscientists a reliable view on local connectivity profiles based on clinical feasible dMRI. This strategy may open new possibilities to, for example, set up repeatable neurosurgical intervention protocols. SPECTRE is complementary to normative approaches for which connectivity information is pooled on group level and warping techniques based on deformable registration of anatomical contrasts are used. For the purpose of demonstration, we concentrated on midbrain structures at the level of the STN, which are particularly important in the context of tractographically guided DBS for Parkinson's disease, OCD and depression. The details of human fiber architecture of these regions are, naturally, not fully understood at this moment. Diffusion MRI at a clinical level can hardly contribute to answer the relevant, open questions about the true effectiveness of DBS due to low resolution and inherent ambiguities (like fanning, crossings) next to the obvious limitations (Maier‐Hein et al., 2017) that apply to tractography (low resolution, false positives/negatives, lack of direction, no synapses shown, no information on transmitters). However, SPECTRE can definitely help with navigation and add structural information and contrast, if the underlying mechanisms are well understood and if the technology proves to be robust.

### 4.1 Robustness

We have chosen a rather smooth, anatomically driven coloring scheme. Neighboring regions on the cortex are similarly colored. It is elusive to trace axons, which are tightly packed in the midbrain and then fan out over the whole neocortex. Uncertainties on the cortical level are expected to be at a (multi‐) centimeter range. Regions like the corona‐radiata are very difficult to disentangle, and the anterior limb of the internal capsule (ICa) is a bottleneck, which is difficult to unscramble. So, we decided to use a smooth and continuous scheme, which is in contrast to others, who use tight segmentation coming from for example, a functional atlas (Ewert et al., 2018). But even with the here proposed rather smooth color coding, single‐shell CSD‐based tractography was already insufficient to produce robust results, the maps are rather noisy and incoherent. Simple tracking based on the principal direction of the diffusion tensor shows a qualitatively different picture, maps are coherent, but unstable and not reproducible. Tensor deflection tractography, multi‐shell CSD and GT‐based tractography lead to stable and mostly reproducible results. But note that these findings cannot be generalized to all brain regions. In areas of higher anisotropy single‐shell CSD has also been proven to give stable results.

We have also seen that susceptibility induced distortions are one of the major problems for the rescan reproducibility of dMRI‐based methods. In fact, SPECTRE can give a direct picture of the underlying features. The midbrain regions are usually highly affected by such distortions due to its proximity to the paranasal sinuses. So, in practice, it is imperative to verify the absence of such strong distortions to allow valid conclusions. By avoiding AC/PC angulations during EPI slice acquisition one can significantly reduce the artifact in the midbrain. For example, using an acquisition plane touching the upper edge of the pons and the lower frontal edge of the corpus callosum mostly eliminates the artifact, although the problems are then shifted to the orbitofrontal prefrontal cortex. Another option is to use left/right as phase encoding direction, however, the induced asymmetry artifact can be quite disturbing.

However, if the image quality is sufficient, the resolution of SPECTRE maps can be actually above the original dMRI resolution, which is a nice feature and similar to what has been shown in tract‐weighted imaging. It can be explained by the tractographic mapping of information from the cortical level, which lives at the scale of 10–20 cm, onto the rather small‐sized midbrain, which has only about 2–4 cm diameter.

### 4.2 Case study

Our case study suggests that focusing the VAT of DBS on the motor region results in an improved motor outcome (improved UPDRS III score and less fluctuations). However, this case only serves as a first demonstration and larger numbers of patients treated are necessary to confirm this observation. We see, however, a large potential in connectivity visualization at the individual level, as a simple overlay on an anatomical MR contrast. For functional stereotactic approaches, the individual motor STN becomes discernible already during the planning phase of DBS electrode placement. Other authors have already shown that the improved outcome of STN DBS is related to its motor connections and that psychotropic side effects coincide with deviated electrode positions and stimulation of limbic STN territories (Petry‐Schmelzer et al., 2019). Using SPECTRE, this connection pattern can now be visualized at high resolution on the level of the individual patient and can be overlayed during three‐dimensional planning of a surgical approach (Coenen et al., 2019) in conventional planning and navigation systems.

### 4.3 Anatomy

SPECTRE shows how the fronto‐parietal‐occipital cortical gradient experiences a twist by 90° (frontal to medial, occipital to lateral) while proceeding ventrally towards the midbrain, where the gradient becomes mostly medial‐lateral with an interruption at the interface between RN and SNR. The interpretation of the fronto‐parietal gradient as limbic‐associative to sensori/motor is quite intuitive, in particular when focusing on the STN. Our results support the hypothesis that there are no clear functionally segregated parts (Alkemade & Forstmann, 2014) in the STN but merely overlapping regions with distinct functions and a continuous and inter‐individually varying gradient of hard‐wiring to the frontal lobe.

It is important in this context that the widely accepted tripartite differentiation of the STN is the result of DBS driven research of the recent years, while the literature—leaning on imaging, tract tracing, transmitter‐immunochemistry—is not in support of segregated parts of the STN (see (Alkemade et al., 2015) for further discussion). In the “classical” and tripartite descriptions (Alkemade et al., 2015) medial/anterior and inferior parts are regarded as limbic and connect prefrontally, especially to frontopolar and orbitofrontal regions. More posterior regions are the prefrontal association regions of the dorsolateral and prefrontal cortex followed laterally by motor parts (Haynes & Haber, 2013). This agrees mostly with the segmentation used by Ewert et al. (2018), while differences exist as to what is meant with a “limbic” sub‐segmentation of the STN. In Ewert's definition this includes the frontomesial cortex (anterior cingulate etc.) and also the cuneus and temporo‐mesial structures like the amygdala.

Based on tractography information the medial STN or medial STN region (MSR, outside the STN proper) in humans are ambiguously defined. Especially in newer literature these regions show some overlap. Tractographically, fibers from the STN proper only reach the DLPFC while fibers reaching the OFC and DMPFC traverse the region medially to the STN (lateral VTA or sometimes “medial STN region” (Temiz et al., 2019)). This is in some contrast to others (Petersen et al., 2019) who appear to show a concentration of these projections on the STN proper (so inside the nucleus) but with some holographically driven modification of tractography results based on an “anatomical consensus.” This work again specifically leans on the same tract tracing studies in the Macaque (Haynes & Haber, 2013) as a good ground truth with respect to STN sub‐segmentation. However, even these tract tracing studies show fibers outside and medial to the STN and therefore might be open to different interpretations like overlapping and redundant connections inside and outside the STN. Like any tract tracing, this work assumes that the hardwiring in the different primate species is similar, a view that is not necessarily shared by all scientists (Jbabdi et al., 2013). It is important to mention that tract tracing studies are inherently limited by the volume and position of injections and by the low number of observations. Therefore, it is not clear if a functional and anatomical segregation of the human primate's STN into distinct parts is necessarily correct (Alkemade et al., 2015; Alkemade & Forstmann, 2014; Keuken et al., 2012).

### 4.4 Applications beyond STN

**FIGURE 9:**
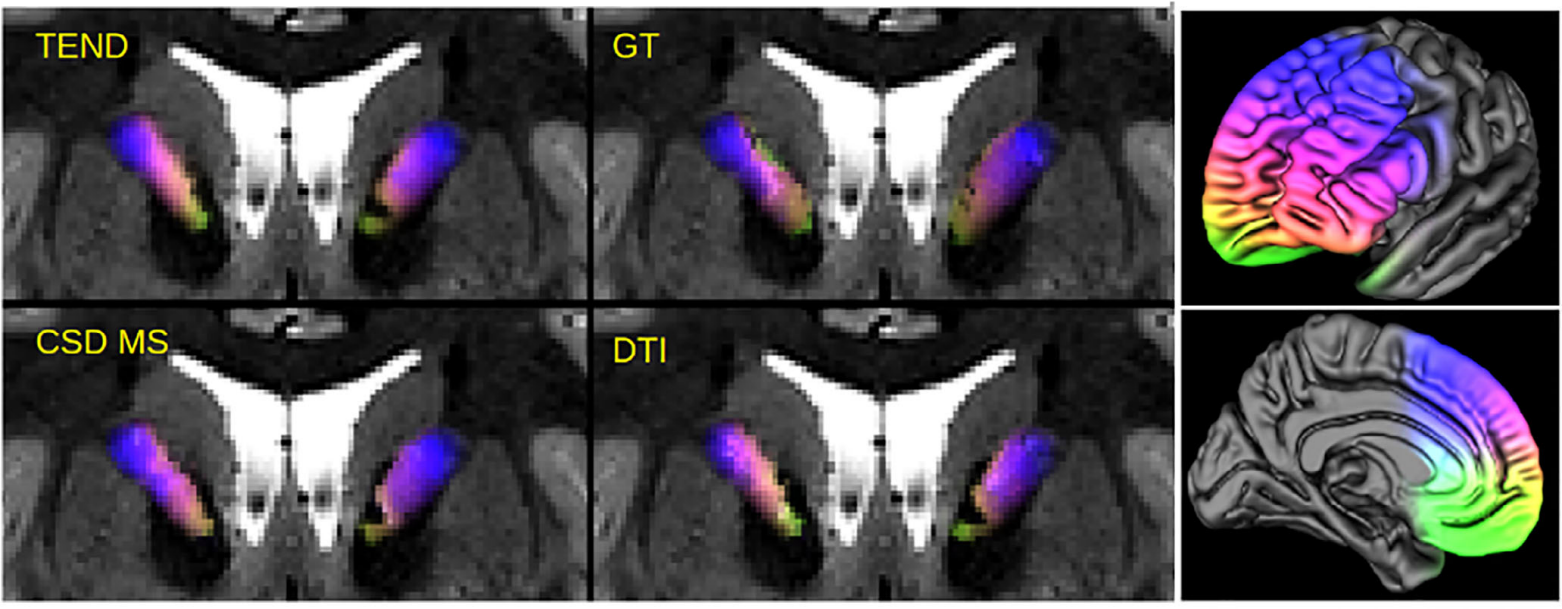
Illustration of SPECTRE application at the ICa with a pure prefrontal coloring (right). SPECTRE maps are shown as an overlay on T2 weighted images. The different approaches (TEND,GT, CSD MS, DTI) agree quite well. The underlying data comes from the example HCP dataset

To demonstrate that SPECTRE is also applicable in other regions of the brain we preliminarily examined the anterior limb of the internal capsule (ICa). The ventral part of ICa is used as a DBS and lesion target in OCD (Liebrand et al., 2019; Liebrand et al., 2020; van Westen et al., 2021) and depression (ALIC target = anterior limb of internal capsule). Our results in Figure 9 show that most projections from OFC and VMPFC (green) are located in the most ventral part of the ICa, while fibers traversing the ICa in dorsal parts stem from dorsolateral DLPFC. Note that there is also a natural gradient in ICa as described in the STN (Figure 9) but in ICa from OFC to DLPFC. These results are in congruence with the discussion about these connections being the most important ones for DBS and stereotactic lesions especially in OCD. Owing to the chosen approach, the ICa rendition here only shows descending connections from PFC. For further discussion on the topic especially with respect to ascending fiber systems in ICa see previous work (Coenen et al., 2020; Nanda et al., 2017; Safadi et al., 2018).

## 5 CONCLUSION

With the aim of providing a more direct view of structural connectivity patterns we have proposed SPECTRE as a novel visualization approach, which joins individual tractographic information with normative and individual anatomical information. It was shown that, if dMRI images are not affected by susceptibility artifacts, SPECTRE maps show reproducible structural connectivity information. This is a prerequisite for any reliable neuroimaging technique which may be regarded, for example, for purposes such as stereotactic planning and other neurosurgical procedures. We have regarded a part of the upper brainstem, the midbrain, which is of special interest in DBS surgery. SPECTRE allows us to appreciate the fronto‐occipital connectivity gradient on the individual level and thereby helps to understand the varying amount of frontopolar contribution of connectivity to the STN and the region just adjacent and medial to it. A clear segregated subdivision of STN anatomy in limbic, associative, and motor parts as proposed before cannot not be found. SPECTRE analysis rather finds a gradient of frontopolar to motor connectivity that varies between individuals and traverses the whole STN. SPECTRE‐based visualization of connectivity patterns as color‐coded information that can immediately be used in any stereotactic planning system might make it a valuable tool for surgery planning and stimulation steering for STN DBS and other regions. Clinical proof and a correlation with clinical effectiveness of DBS are the focus of current research.

## CONFLICT OF INTEREST

The authors declare no conflicts of interest.

## CODE AVAILABILITY

Upon acceptance the MATLAB code and C++ code, which was used to create the proposed SPECTRE maps, will be available in a public code repository (Bitbucket https://bitbucket.org/reisert/spectre/src/master/).

## Supporting information


**Appendix S1**. Supporting Information

## Data Availability

The data that support the findings of this study are available from the corresponding author, Marco Reisert, upon reasonable request.
