# Supplementary material for: SPECTRE—A novel dMRI visualization technique for the display of cerebral connectivity
Source: Hum Brain Mapp. 2021 Feb 27;42(8):2309–21. doi: 10.1002/hbm.25385 (PMC8090769; doi:10.1002/hbm.25385)
Supplement: Supplementary file 1 — Appendix S1. Supporting Information [file HBM-42-2309-s001.docx]

**Appendix S1**

**Computations of fiber orientation distributions (FODs)**

It is known that the anisotropy of the diffusion signal is rather low particularly in the midbrain and the adjacent basal ganglia areas. This makes the determination of the FODs in these regions difficult and more susceptible to imaging noise. We considered here five different approaches, which will demonstrate these difficulties and how to cope with them by using prior knowledge about the nature of fibrous tissue. Besides ordinary tensor estimation the following approaches were applied: CSD, multi-shell CSD, spatially regularized CSD and tract orientation distributions (TODs) derived by global tractography^18^ (GT). The experienced reader may wonder why we did not use the streamlines reconstructed by GT directly for rendering of SPECTRE maps. The main reason is that the number of reconstructed streamlines is by far too small to generate high resolution SPECTRE maps. Thus, to still gain advantage of the robustness of GT in regions with low anisotropy, we used the TODs of GT as basis for tractography. Moreover, due to the generative nature of GT (global tractography searches for streamline configurations, which explain the observed data as best as possible), the TODs of GT are just fiber orientation distributions (FOD) in its original definition. That is, GT may also be interpreted as a regularized CSD approach, where the reconstructed streamlines are rather a side product.

**Principal Tensor Direction (DTI):** As the simplest approach we used the principal direction of the diffusion tensor, which is estimated by an ordinary least-squares estimate (for multi b-shell protocols the lowest b-shell is used for estimation). Therefore, the full diffusion tensor is estimated and the eigenvector corresponding to the maximum eigenvalue is selected.

**Constrained Spherical Deconvolution (CSD):** Further, a straight-forward CSD [(Tournier et al., 2007)](https://www.zotero.org/google-docs/?6ejEQz) is used with the standard parameters (https://www.mrtrix.org/). To determine the fiber response function *dwi2response* was used with Tournier’s algorithm [(Tournier et al., 2013)](https://www.zotero.org/google-docs/?Vs05Hp). The three most prominent directions of the FODs are extracted for tractography (sh2peaks).

**Multi-Shell Constrained Spherical Deconvolution (CSD MS):** According to Jeurissen et al [(Jeurissen et al., 2014)](https://www.zotero.org/google-docs/?m1KZZW) a multishell CSD approach was examined, where the tissue model is composed of three compartments (https://www.mrtrix.org/). To determine the fiber response function *dwi2response* was used with Tournier’s algorithm [(Dhollander et al., 2018)](https://www.zotero.org/google-docs/?CGQbi0). The three most prominent directions of the FODs are extracted for tractography (sh2peaks).

**Fiber Continuity Regularized CSD (fcCSD):** Here, we follow a PDE-based global estimation of the FOD-field based on the fiber continuity assumption [(M. Reisert & Kiselev, 2011)](https://www.zotero.org/google-docs/?0KaXNI). We use the Spherical Harmonic implementation as proposed in [(Marco Reisert & Skibbe, 2013)](https://www.zotero.org/google-docs/?J8JAHT) and available as part of the STA-toolbox.

**Global Tractography (GT):** As already described above as a third approach we used the tract orientation distributions (TOD) of the global algorithm [(Marco Reisert et al., 2011)](https://www.zotero.org/google-docs/?Rdvd7B) as a proxy for the fiber orientation distributions. We used the standard dense parameter setting as provided by the toolbox Fiber & Diffusion Toolbox. (https://www.uniklinik-freiburg.de/mr-en/research-groups/diffperf/fibertools.html)

After tractography the TODs were rendered and the 3 most prominent directions were used for probabilistic tracking (see below).

**Probabilistic Integration based on Peak Distributions**

The probabilistic streamline integration closely followed the work of Behrens [(Behrens et al., 2007)](https://www.zotero.org/google-docs/?ZROJmy), similar to FSL's probtrackx. The evolution equations are

$x_{n+1}= x_{n}+\alpha\frac{d_{n}}{|d_{n}|}$ (1)

$$d_{n+1}=argmax_{v\in F(x_{n})} (v\cdot d_{n}) + sN$$

where$x_{n}$ and $d_{n}$ are current position and propagation directions in the n-th step,$\alpha$the step-width and s the noise factor and $N$ a Gaussian distributed random vector with standard deviation of 1. The set $F(x)$ contains all possible tracking directions in voxel $x$ (including both signs, so overall 2*3=6 directions). In this formulation, the noise component is related to the magnitudes of $v\in F(x)$, which represent, depending on the method for their estimation, different quantities. In ordinary CSD it is more related to apparent fiber density, for GT it is a tract density, and for DTI it is just the FA value. To become independent of the absolute magnitudes a simple option is to normalize the directions within a voxel by their norm, which we followed in the experiments (see below for a discussion of this decision). Note that for DTI the set $F(x)$ just refers to the one-member set containing the main eigenvector. The algorithm is then very close to a probabilistic version of the FACT algorithm (fiber assignment by continuous tracking) as provided in the Diffusion Toolkit (http://www.trackvis.org/dtk/). The seed position$x_{0}$is randomly chosen within the seed voxel uniformly. Streamlines are terminated when they touch the border of the white matter mask, which is provided by CAT12.

**Influence of Normalization and Comparison to iFOD2 Peak Distributions**

The interested reader may ask, how the proposed tracking approach relates to other established methods (like iFOD2) and what the differences are. Also the influence of the peak normalization approach described above has to be investigated. Therefore, we used MRtrix tckgen with default iFOD2 parameters. The resulting tracts (10 million) were then aggregated to SPECTRE maps using the same track-weighting scheme as described in the method section (at 1mm resolution). Additionally, we tested a ‘non-normalized’ version of the approach described in eqn (1), i.e. the directions $v\in F(x)$ are not scaled to unity, but still contain the magnitude coming from the original FOD. In Figure 10 the corresponding SPECTRE maps are shown. For ordinary CSD we observe that the non-normalized approach is very different compared to the normalized, while the normalized version is much closer to what we get when using iFOD2 as a tracking approach. For the non-normalized approach the streamlining in the low anisotropy STN/Ruber region tends to be very random and streamlines stay quite long in non-colored regions, which leads to a low ‘SPECTRE-signal’ in these regions. In Figure 10d) the non-normalized iFOD2 approach with a different brightness windowing is presented, which shows that there is indeed some remaining structure in the ‘dark regions’ visible, but with much less magnitude. For GT the visual differences between un-normalized and normalized tracking are relatively small, which is mainly due to the spatial coherence of the peak directions and the lower variance of the peak magnitudes.


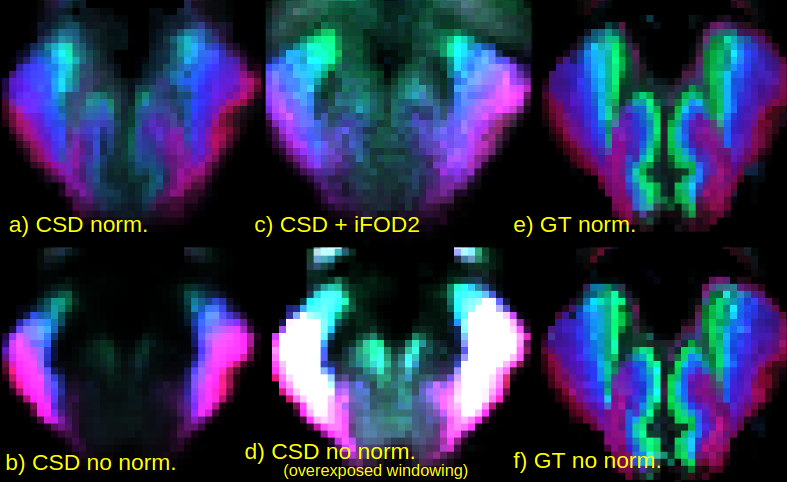


**Figure 10) Comparison of tracking approaches on the considered HCP subject.** a,b) SPECTRE maps based on MRtrix peak orientations. The tracking algorithm in a) uses peak amplitudes, which are normalized prior to adding the random perturbation. In b) the peak magnitudes are corresponding to FOD magnitudes at the peak. Plot d) shows the same as b) but with a different coloring window. In c) SPECTRE maps are shown, which use iFOD2 as the underlying tracking mechanism. Finally in e) we show for reference results based on GT as shown throughout the paper with normalization and in f) without normalization.

**Probabilistic Integration based on Tensor Deflection**

As an alternative we followed a probabilistic version of the tensor deflection approach [(Lazar et al., 2003)](https://www.zotero.org/google-docs/?Jw3fmJ) (TEND). The propagation equations are given as follows

$$x_{n+1}= x_{n}+\alpha\frac{d_{n}}{|d_{n}|}$$

$d_{n+1}=d + \sigma N$ where $d=D(x_{n}) d_{n} / | D(x_{n}) d_{n}|$

and where again$x_{n}$ and $d_{n}$ are current position and propagation directions in the n-th step,$\alpha$the step-width and $s$ the noise factor. The $D(x_{n})$ denotes the normalized (by its Froebenius norm) diffusion tensor at position $x_{n}$. Throughout the experiments we fixed the step size$\alpha$to 1mm. The noise factor was varied (see details in the main text).
